# Outcomes of descemet stripping automated endothelial keratoplasty using imported donor corneas

**DOI:** 10.1186/s12886-017-0436-0

**Published:** 2017-04-05

**Authors:** Kaevalin Lekhanont, Kavin Vanikieti, Nutthida Nimvorapun, Varintorn Chuckpaiwong

**Affiliations:** Department of Ophthalmology, Ramathibodi Hospital, Mahidol University, Rama VI Rd., Rajathevi, Bangkok, 10400 Thailand

**Keywords:** Descemet stripping automated endothelial keratoplasty, Outcomes, Imported donor corneas

## Abstract

**Background:**

The lack of development of local donor tissue acquisition in several regions of the world has resulted in the necessity of performing keratoplasty with imported donor corneas. The greatest concern about the use of donor corneas supplied by foreign eye banks is the effect of the increased donor death-to-operation time which inevitably occurs during the tissue recovery, tissue processing, and tissue transfer between the countries. The purpose of this study was to report the outcomes of descemet stripping automated endothelial keratoplasty (DSAEK) using imported donor corneas.

**Methods:**

This retrospective, non-comparative case series investigated the outcomes of the 102 consecutive DSAEK procedures using imported donor corneas performed at a single university-based hospital between August 2006–2014. The main outcome measures were postoperative best-corrected visual acuity (BCVA), endothelial cell density (ECD), and complications.

**Results:**

The mean death-to-operation time was 9.52 ± 1.48 days (range, 8–13). The mean preoperative ECD was 2761 ± 285 cells/mm^2^. Fuchs’ endothelial dystrophy was the predominant indication for grafting. The mean follow-up duration was 65.3 months. Ninety-three eyes had improved vision postoperatively (91.18%). BCVA unchanged in 3 eyes due to preexisting macular scar and advanced glaucoma. Primary graft failure occurred in 6 eyes (5.88%). Of the 93 eyes with improved BCVA, 100% had their best corrected vision within the first 1 year. The mean ECD at 6, 12, 24, 36, and 60 months after surgery was 1762 ± 294 cells/mm^2^, 1681 ± 284 cells/mm^2^, 1579 ± 209 cells/mm^2^, 1389 ± 273 cells/mm^2^, and 1251 ± 264 cells/mm^2^ respectively. The mean ECD loss at 6 months, 1 year, 2 years, 3 years, and 5 years after surgery was 36.2%, 39.1%, 42.8%, 49.7%, and 54.7% respectively. The most common complication was graft detachment/dislocation (10.78%). There were no cases of any postoperative infection.

**Conclusions:**

DSAEK with imported donor corneas provides rapid and good visual rehabilitation. The percentages of endothelial cell loss were comparable to those achieved in Western series using domestic corneas in which fresher tissues were available for transplantation.

## Background

Descemet stripping automated endothelial keratoplasty (DSAEK) has become the first-choice and most popular method for the treatment of corneal endothelial diseases. DSAEK appears to be superior to penetrating keratoplasty (PK) in terms of rapid visual recovery and refractive stability, surgically induced astigmatism, higher order aberrations, structural strength of the eye, postoperative surface keratopathy, wound and suture-related complications, rates of transplant rejection, and intraoperative and late choroidal hemorrhage risk [1, 2]. However, graft survival remains an important issue [1, 3]. Most previous studies found that 1- and 2-year endothelial cell losses were greater following DSAEK, consistent with more donor tissue manipulation in DSAEK [2–7]. Fortunately, the graft success rate and endothelial cell loss in the surviving grafts were comparable at 3 and 5 years for DSAEK and PK procedures [8, 9]. Nonetheless, acceleration of cell loss may lead to earlier onset of late endothelial failure and ultimate graft decompensation [1].

The shortage of donor corneas is a major issue worldwide. Many countries, including Thailand, face this problem as cornea donation is not allowed or encouraged in some cultures due to the religious faith and traditional beliefs. To overcome the insufficient donor supply, we have been using both donor corneas from oversea eye banks and domestic donor corneas for keratoplasty. Excellent efficacy and safety of PK utilizing foreign donor corneas have been previously demonstrated [10–12]. Also, there has recently been a report showing the satisfactory outcomes of DSAEK performed with internationally shipped, precut donor corneas [13]. The study was conducted in Japan and the approximate flight duration time from the United States to Narita plus the overland freight transportation time was only 17–18 h. However, the flight between the United States and Bangkok and the overland transit takes about 48 h. This raises additional critical concerns regarding the suitability of foreign donor corneas for the Thai population, corneal graft survival associated with imported tissue, the potential loss of endothelial cell density (ECD) and viability secondary to prolonged donor death-to-operation time, and unexpected consequences caused by the transportation over longer distances such as tissue damage or the growth of infectious organisms. The purpose of this study was to assess the efficacy and safety of DSAEK using internationally shipped donor corneas in Thailand.

## Methods

### Study design

This was a single-center, retrospective, non-comparative interventional case series, analyzing the outcomes of DSAEK using imported donor corneas in patients with corneal decompensation due to endothelial failure. Clinical data were obtained from a review of the medical records.

### Participants

One hundred and two eyes of 95 patients with endothelial decompensation who underwent DSAEK at Ramathibodi Hospital, Bangkok, Thailand, from August 2006 and August 2014, with a minimum follow-up of 24 months were recruited into this trial. Patients with the accompanying risks of anterior segment abnormalities or further surgeries unrelated to the DSAEK procedure that may influence postoperative endothelial cell survival were excluded from the study. The study was approved by the ethics committee of Mahidol University School of Medicine and adhered to the tenets of the *Declaration of Helsinki*. All patients were informed regarding the advantages, disadvantages, and potential complications of this surgery and emphasized the importance of postoperative care and need for regular follow-up and monitoring after surgery. Written informed consent was obtained from each individual prior to enrollment in this study.

### Interventions

All donor corneas were provided by Eye Bank Association of America (EBAA)-accredited facilities in the United States. Each tissue met EBAA minimum standards of donor age, ECD, and death-to-preservation time. The corneas were transported as corneoscleral buttons housed within corneal viewing chambers filled with Optisol-GS (Bausch & Lomb, Rochester, NY). The viewing chambers were sealed with plastic wrap, packed in a plastic bag, and positioned in a soft foam block to prevent excessive movement. The foam blocks were again sealed in a plastic bag and placed into generic, thick-walled, expanded polystyrene foam shipping containers with wet ice. The packages were sent from the United States to Bangkok Suvarnabhumi International Airport and then transferred to Ramathibodi Hospital via FedEx. The containers were maintained at 2 °C–8 °C in validated shipping containers throughout the entire shipment process. Upon arrival at Ramathibodi Hospital, a technician collected the container and completed a tissue arrival check, including the date and time of arrival, condition of the shipping container, number and status of the ice blocks, number of donor tissues, and status of each viewing chamber. Also, the technician matched and confirmed the documentation accompanying each tissue, and delivered it to the operative room (OR) charge nurse. The tissue and its package must have been received in good condition otherwise it would have been considered not suitable for transplantation. The OR charge nurse placed it in the temperature-controlled refrigerator. On the day of surgery, the tissue was removed from the refrigerator, reevaluated whether it was well suited for transplantation, and allowed to warm to room temperature in the operating theatre for 30–60 min prior to use.

Two novice surgeons (KL, VC) performed the DSAEK procedures under either local or general anesthesia. The donor tissue was dissected with a microkeratome in all cases using the Moria ALTK system (Moria Surgical, Antony, France). Tissue preparation was done by the operating surgeon in 33 eyes and by an US eye bank technician (pre-cut tissue) in 69 eyes. Descemet’s membrane and diseased endothelium were stripped from the planned graft area using the reverse Sinskey hook and a DSAEK stripper. Venting incisions were made to improve graft adherence. A 4.5–5 mm clear corneal incision was created using a 3.0 mm—wide slit knife for graft insertion. The donor cornea was cut to a diameter of 7.5–9.0 mm, depending on the recipient corneal diameter. The trephined donor posterior lenticule was inserted into the recipient anterior chamber using Busin glide (Moria, Doylestown, PA). Once the donor graft was properly unfolded and well centered, it was pressed up against the recipient stroma with air. After the anterior chamber was filled completely with air for 10 min, the air bubble was then reduced to prevent pupillary block. Beginning with case 9, the inferior peripheral iridectomy was performed prior to donor insertion to allow leaving nearly a full air fill in the anterior chamber at the end of surgery without producing pupillary block.

Postoperatively, each patient received topical corticosteroids (1% prednisolone acetate) 8 times daily and antibiotics 4 times daily for the first 2 weeks. They were gradually tapered at the discretion of the operating surgeons to 1 drop per day dosing, continued indefinitely. Steroid responders received intraocular pressure (IOP)-lowering medications. Also, prednisolone acetate 1% was changed to fluorometholone as needed. Patients were examined daily until the residual air bubble did not cover the pupil when the patient sat up and corneal re-epithelialization was complete and then discharged from the hospital. Follow-up evaluations were performed at 0.5, 1, 3, 6, 9, and 12 months after surgery and then every 6 months thereafter.

### Outcome measures

The primary outcome measures included postoperative best-corrected visual acuity (BCVA), endothelial cell density (ECD), and complications. The secondary outcome was the relationships of the postoperative ECD with donor age, death-to-preservation time, death-to-operation time, and baseline donor ECD. Preoperative donor ECD was evaluated by provider eye banks in the United States using specular microscopy. Postoperative ECD was determined by noncontact specular microscopy (EM-3000; Tomey, Nagoya, Japan) in our clinic at 6 months, 1, 2, 3, and 5 years after surgery. The central corneal endothelium was photographed using the auto-alignment and auto-shot functions of the instrument. The best-quality image is automatically selected and analyzed by the preinstalled software of the instrument (Cell and Layer Analyser; Tomey). The number of analyzed corneal endothelial cells and cell density were automatically calculated using the fixed-frame counting method in an area set to 100 μm^2^ without manual cell border correction. At least 50–100 cells were required to be examined for each image and the postoperative ECD was recorded. Percentage of endothelial cell loss was calculated by subtracting postoperative ECD from preoperative ECD and dividing by preoperative ECD, and then multiplying by 100. Endothelial polymorphism and polymegathism analyses were not performed. Anterior segment OCT was also not performed postoperatively as a result of the unavailability of the equipment at that time. Patients having complications received intensive care in collaboration with other subspecialists.

### Statistical analysis

Statistical analyses were performed with the statistical software package STATA version 11.1 (Stata Corp, College Station, Texas, USA). Continuous data were expressed as either mean ± standard deviation (SD) or median and range, depending on the normality of distribution. These were compared using independent t test or Mann–Whitney U test respectively. Categorical data described as frequency and percentage, were analyzed with Chi-square or Fisher’s exact tests. Associations between postoperative ECD at 5 years and various donor or recipient characteristics were evaluated using linear regression analysis. Multiple regression analysis was performed for only variables that were statistically significant by univariate analysis. *P*-values less than 0.05 were considered to be statistically significant.

## Results

Donor characteristic data including donor age, death-to-preservation time, death-to-surgery time, residual stromal bed thickness after microkeratome cutting, and ECD are summarized in Table 1. Data were not available on all parameters for all donor tissues due to either no information provided from the Eye Banks or missing data. Death-to-operation time was greater than or equal to 8 days in all corneas. An 8.0- to 8.5-mm donor disc was used in 94.12% of cases. Table 2 presents patient demographics and indication for surgery. The mean recipient age was 64.21 ± 19.56 years, 58.82% were female, and fuchs’ endothelial dystrophy and pseudophakic bullous keratopathy (PBK) were the predominant indications for grafting. Forty-two DSAEK operations (41.18%) were performed in combination with phacoemulsification and posterior chamber intraocular lens implantation (IOL). Most patients had no preexisting glaucoma. Only 6 patients had undergone prior glaucoma surgeries. The mean follow-up duration was 65.3 months (range, 24 to 96 months).

**Table 1:** Donor Characteristics of 102 imported donor corneas for Descemet stripping automated endothelial keratoplasty

Characteristics	Number
Mean age (year)	63.85 ± 15.94 (range, 20–75)
Death-to-preservation time (hours)	12.52 ± 7.45 (range, 5–20.45)
Death-to-operation time (days)	9.52 ± 1.48 days (range, 8–13)
Endothelial cell density (before precut) (cell/mm^2^)	2761 ± 392 (range, 2361–3015)
Central corneal thickness (μm)	585.53 ± 87.81 (range, 534–685) (*n* = 85)
Graft thickness (post-cut) (μm)	142.37 ± 65.29 (range, 96–225) (*n* = 65)
Graft diameter, number (%)	
- 7.5 mm	3 (2.94)
- 8.0 mm	47 (46.08)
- 8.5 mm	49 (48.04)
- 9.0 mm	3 (2.94)

**Table 2:** Recipient Characteristics of 102 eyes undergoing Descemet stripping automated endothelial keratoplasty

Characteristics	Number
Mean age (year)	64.21 ± 19.56 (range, 5–90)
Sex	Female 60 (58.82%) Male 42 (41.18%)
Diagnosis, number of eyes (%)	
- Fuchs’ endothelial dystrophy	46(45.10)
- Pseudophakic bullous keratopathy	32 (31.37)
- Laser iridotomy induced corneal decompensation	15(14.71)
- Iridocorneal endothelial (ICE) syndrome	3 (2.94)
-Cytomegalovirus (CMV) endotheliitis	3 (2.94)
- Congenital hereditary endothelial dystrophy (CHED)	2 (2.78)
- Unknown cause	1 (1.39)
Preoperative lens status, number of eyes (%)	
- Pseudophakic	57 (55.88)
- Phakic	45 (44.12)
Combined procedure, number of eyes (%)	
- Phacoemulsification with intraocular lens implantation	42 (41.18)
Glaucoma status, number of eyes (%)	
- No preexisting glaucoma	76 (74.51)
- Medically controlled without prior surgery	20 (19.61)
- Prior glaucoma surgery	6 (5.88)

**Fig. 1:**
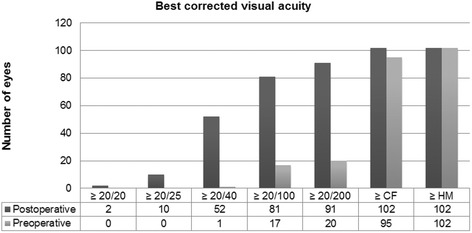
Preoperative and postoperative best-corrected visual acuity of 102 patients undergoing descemet stripping automated endothelial keratoplasty with imported donor corneas

Overall, BCVA in 102 eyes at 6 months improved to 20/63 (range 20/20 to 20/200), representing an average gain of 5 Snellen lines from preoperative vision (*P* < 0.01). Preoperative BCVA was ≤20/200 in 80 eyes (78.43%) and ≤20/40 in 101 eyes (99.02%). Ninety-three eyes had improved vision postoperatively (91.18%). BCVA unchanged in 3 eyes (2.94%) due to preexisting macular scar (2) and advanced glaucoma (1). Six eyes (5.88%) experienced a decline in vision secondary to primary graft failure. Of the 93 eyes with improved BCVA, 100% had their best corrected vision within the first 1 year. Postoperatively, 9.80% achieved a BCVA of ≥20/25, 50.98% achieved ≥20/40, and 79.41% achieved ≥20/100 by the last follow-up (Fig. 1).

The mean preoperative ECD was 2761 ± 285 cells/mm2 (range, 2361–3015). Among eyes examined with clear grafts, the mean ECD at 6, 12, 24, 36, and 60 months after surgery was 1762 ± 294 cells/mm^2^, 1681 ± 284 cells/mm^2^, 1579 ± 209 cells/mm^2^, 1389 ± 273 cells/mm^2^, and 1251 ± 264 cells/mm^2^ respectively. The mean ECD loss at 6 months, 1 year, 2 years, 3 years, and 5 years after surgery was 36.2%, 39.1%, 42.8%, 49.7%, and 54.7% respectively (Table 3). From univaraite and multivariate analyses, 5-year ECD was not significantly correlated with donor age, death-to-preservation time, death-to-surgery time, and graft diameter. The baseline donor ECD was the only variable positively significantly associated with 5-year ECD (*P* = 0.02).

**Table 3:** Postoperative endothelial cell density and cell loss after Descemet stripping automated endothelial keratoplasty

Time	Endothelial cell density (cells/mm^2^)		Endothelial cell loss
	Mean ± SD (range)	Number of eyes	Percentage
Baseline	2761 ± 285(2361–3015)	102	
6 months	1762 ± 294 (1084–2263)	96	36.2 (±13.9)
1 year	1681 ± 284 (997–2115)	92	39.1 (±12.4)
2 years	1579 ± 209(753–2048)	85	42.8 (±13.1)
3 years	1389 ± 273 (729–1893)	79	49.7 (±14.7)
5 years	1251 ± 264 (683–1589)	72	54.7 (±13.9)

Postoperative complications after DSAEK surgery are shown in Table 4. The most common complication was graft detachment (11 eyes, 10.78%). Three eyes experienced donor lenticule dislocation into the anterior chamber on the first postoperative day and required a second surgery of graft repositioning and air bubble replacement. One of these 3 eyes had had an eccentric mis-cut with the donor trephine due to a large graft size (9.0 mm) used and an erroneous reliance by the surgeon on the center of the donor cornea after precutting the tissue in the eye bank. The 1.5-mm maximum width and 4-clock hour meridian full-thickness edge was not trimmed before insertion to avoid the potential endothelial cell insufficiency and attendant excessive endothelial trauma. This patient suffered recurrent dislocation on the second postoperative day and needed a second repositioning and rebubbling of the graft for it to become attached. Despite the successful graft reattachment, the graft remained edematous and was considered iatrogenic donor failure. The patient refused to have a repeat DSAEK because of extremely old age and difficulty in postoperative patient positioning so that she underwent a new PK. The other 2 eyes were 1 post-trabeculectomy and 1 post-PK eyes which were unable to maintain air in the anterior chamber because the air was misdirected into the bleb in post-trabeculectomy eye and absorbed too soon in the post-PK eye. After the first graft repositioning, the grafts were adherent and clear during follow-up visits in these 2 eyes. The remaining 8 eyes with graft detachment were successfully reattached with a rebubble procedure on the next day in a minor surgical suite.

**Table 4:** Postoperative complications after Descemet stripping automated endothelial keratoplasty

Complications	Number of eyes (%)
Graft dislocation/detachment	11 (10.78)
Primary graft failure	6 (5.88)
Pupillary block	6 (5.88)
Late graft failure	6 (5.88)
Graft rejection	5 (4.90)
Secondary angle closure glaucoma	3 (2.94)
Intraocular lens dislocation	1 (1.39)

Primary graft failure occurred in 6 eyes and one of them was the eye with eccentric mis-cut. Two eyes required reinsertion of the donor lenticule into the anterior chamber. Two eyes had a thin, precut donor lenticule, causing more tissue manipulation during transfer to the glide. Another eye underwent uneventful DSAEK using an eye bank-prepared precut cornea. Of the 6 eyes with primary graft failure, the donor grafts were surgeon-prepared in 4 eyes and technician-prepared in 2 eyes. Five patients developed episodes of endothelial graft rejection and all were treated with systemic and topical corticosteroids. Graft rejection was observed within 1 year post-surgery in 2 eyes and after 1 year in 3 eyes. Six eyes experienced late graft failure; 2 was post-rejection, 1 was post-additional IOL surgery, and 3 had no obvious precipitating factor. Pupillary block occurred in 6 eyes without surgical peripheral iridectomy at the time of DSAEK. It was relieved by partial removal of the air bubble or peripheral iridectomy. There was no pupillary block occurred in any cases after inferior peripheral iridectomy was adopted as a routine policy. One eye having a diagnosis of PBK secondary to complicated phacoemulsification with posterior capsular tear experienced posterior IOL dislocation after DSAEK. It possibly resulted from unrecognized small zonular dialysis or undetected placement of a haptic in the capsular bag. The IOL was successfully repositioned by a retinal specialist; however, the graft eventually failed 13 months following IOL surgery.

Corneoscleral rim cultures were obtained in all cases and none had a positive donor rim culture. There were no cases of infectious keratitis or endophthalmitis.

## Discussion

The lack of development of local donor tissue acquisition in several regions of the world has resulted in the necessity of performing DSAEK with internationally shipped donor corneas. The greatest concern about the use of donor corneas supplied by foreign eye banks is the effect of the increased donor death-to-operation time which inevitably occurs during the tissue recovery, tissue processing, and tissue transfer between the countries. In addition, the shipping-related vibration effects and the changes in the air pressure caused by air transport might affect the quality of the donor corneas [13, 14]. Thus, outcomes of DSAEK using imported donor corneas with longer-distance transportation have yet to be determined.

Our study demonstrated that despite having longer transportation time, there was a satisfactory BCVA improvement in 91.18% of patients after DSAEK using imported donor corneas. Additionally, although the postoperative mean ECD at 6 month, 1, 2, 3, and 5 years of this study was lower than those of the Japanese report and previous studies of domestically shipped corneas, the percentage of endothelial cell loss at each time point in our study was similar to those of such studies [6–8, 13]. Since the mean preoperative ECD before precutting and the percentage of postoperative endothelial loss in this study were not obviously different from those of previous studies, the lesser postoperative mean ECD at all follow-up time points might be due to the lower number of ECD just prior to surgery. However, this could not be confirmed because the eye bank specular microscope was unavailable at our institute so that changes in the ECD secondary to precutting process and overseas transportation were not assessed. Although there is no considerable loss of donor endothelial cells during the first week of storage in Optisol media [13, 14], the ECD would be expected to decrease steadily and probably drop below the level of 2200 cells/mm^2^ after the 10th post-harvest day [15]. In this study, death-to-operation time was greater than or equal to 8 days in all corneas. Thus, re-evaluating tissue stored in Optisol-GS for more than 7 days by repeat specular microscopy prior to using the tissue may be recommended if the equipment is provided.

With respect to the relationship between donor tissue storage time and the probability of endothelial survival, there has still been conflicting evidence in the literature. A significant correlation between prolonged storage time (> 7 days) and increased risk of graft failure has been reported in PK with imported donor corneas [16]. Nevertheless, this outcome might be caused by the use of tissues for high-risk PK. Recent studies showed the opposite finding that in a series of low-risk PK, there was no correlation between preservation-to-surgery time and graft survival probability [10, 17]. As for DSAEK, previous studies from Japan found that there was a statistically significant decrease in ECD (2.3–3.79%) associated with overseas transportation of corneal grafts, however, all graft had an ECD before surgery of greater than 2000 cells/mm^2^ and were available for DSAEK [13, 14]. The clinical outcomes of DSAEK with internationally shipped precut donor corneas were also acceptable and that the endothelial cell loss caused by transportation did not appear to influence the clinical results [13]. No association between the storage time and the decline in ECD was observed as well [14, 18]. Furthermore, a wide range of values for the precut donor characteristics such as donor age, death-to-transplantation time, precutting-to-transplantation time, and donor lenticule thickness resulted in excellent adhesion of the tissue and clear grafts [19]. Also, none developed primary graft failure in previous studies [14, 19]. These findings might be explained by the relatively short death-to-surgery time in all patients in prior studies. The longest storage time in previous studies either using internationally (Japanese series) or domestic shipped precut donor corneas (US series) was not greater than 8 days. Meanwhile, the donor corneas in this current study had 4.92 extra days of storage time compared to the Japanese report [13] and the storage time of the shortest stored corneas was 8 days (range, 8–13). Even though the overall results were favorable and no significant correlation between death-to-surgery time and 5-year ECD was seen, there were 6 cases of primary graft failure (5.88%), 11 cases of graft detachment (10.78%), and 3 cases of late graft failure without precipitating factor (2.94%) in our series which is higher than those found the Japanese series. Possible explanations could be both surgical technique and donor characteristics issues. Since the authors were not already experienced DSAEK surgeons, some cases were during our learning curve. The primary graft failure was more common in the earlier cases and at least half of these (3 cases) were iatrogenic (one mis-cut and 2 with more tissue manipulation). This presumably is unrelated to the shipping and storage time. However, as the surgeons were past their learning curve plus the surgical technique used and the rates of endothelial cell loss in this study are similar to those of other studies, the donor characteristics of the longer tissue storage time may be another reason. The baseline donor ECD was also the variable positively significantly associated with 5-year ECD. Therefore, despite the fact that postoperative complications in DSAEK are not tissue dependent, but rather technique dependent; and the higher preoperative endothelial cell counts may not protect against the postoperative event of primary graft failure or graft dislocation, nor may they ensure a higher postoperative cell count than tissues with lower cell counts before surgery in corneas stored in Optisol-GS for ≤7 days [5, 18], using the donor corneas with high cell counts along with performing a flawless surgery may be necessary if a donor tissue for DSAEK has been stored for more than 7 days. Moreover, minute surgical trauma might have an over detrimental effect when using the vulnerably old donor corneas with low cell counts.

It is interesting that the endothelial cell density was slightly changed between 36 and 60 months. This corresponds to the earlier finding that long-term endothelial cell loss in DSAEK patients plateaus more quickly than in those who undergo PK [7, 8]. The impact of less long-term postoperative endothelial cell loss after PK with large donor diameter might be applicable in DSAEK [20], as grafts larger than 8.0 mm in diameter were commonly used in our DSAEK patients. However, the results were conditional on surviving grafts. The ECD of patients with graft failure could not been measured and calculated because of poor quality scans. Hence, there was the potential bias owing to dropout of eyes from the analysis data set when grafts failed [20].

Considering the occurrence of other postoperative complications, graft rejection was found in only 4.9%, secondary angle closure glaucoma in 2.94%, and no infectious keratitis or endophthalmitis was seen in this series. This observation suggests that DSAEK using foreign donor corneas with prolonged storage time have a good safety profile, resembling those using domestic donor corneas [21].

The limitations of this study included the small and heterogeneous patient populations, 2 different surgical techniques used (precut and surgeon-cut), short follow-up time, no comparison of endothelial cell loss between the early and later cases, and inherent shortcomings in any retrospective study.

## Conclusions

In summary, the outcomes of DSAEK performed with internationally shipped donor corneas were acceptable and the percentages of endothelial cell loss were comparable to those achieved in Western series using domestic corneas in which fresher tissues were available for transplantation. This study also supports the efficacy and safety of imported donor corneal tissues stored greater than 1 week and up to 13 days. Cornea donation from overseas eye banks may be of substantial benefit to patients in countries with inadequate donor cornea supply.
